# Quality of Life, Depression, Anxiety and Coping Strategies after Heart Transplantation

**DOI:** 10.21470/1678-9741-2017-0029

**Published:** 2017

**Authors:** Fulvio Bergamo Trevizan, Maria Cristina de Oliveira Santos Miyazaki, Yasmin Lima Witzel Silva, Christiane Maia Waetman Roque

**Affiliations:** 1 Faculdade de Medicina de São José do Rio Preto (FAMERP), São José do Rio Preto, SP, Brazil.; 2 Department of Psychology of Faculdade de Medicina de São José do Rio Preto (FAMERP), São José do Rio Preto, SP, Brazil.; 3 Fundação Faculdade Regional de Medicina de São José do Rio Preto (FUN-FARME), São José do Rio Preto, SP, Brazil.; 4 Hospital de Base (HB), São José do Rio Preto, SP, Brazil.

**Keywords:** Quality of life, Heart Transplantation, Anxiety, Depression, Psychological Adaptation

## Abstract

**Introduction:**

Heart transplantation is the therapeutic procedure indicated to increase the survival of patients with refractory heart failure. Improvement in overall functioning and quality of life are expected factors in the postoperative period.

**Objective:**

To identify and evaluate mental disorders and symptoms, such as depression and anxiety, quality of life and coping strategies in the post-surgical situation of heart transplantation.

**Methods:**

A cross-sectional, quantitative study with patients who have undergone heart transplantation. Participants answered to the Sociodemographic Questionnaire, Beck Depression Inventory (BDI-II), Beck Anxiety Inventory (BAI), MINI International Neuropsychiatric Interview, Escala Modos de Enfrentamento de Problemas (Ways of Coping Scale) (EMEP) and World Health Organization Quality of Life-BREF (WHOQOL-BREF). For data analysis, the significance level was considered *P*≤0.05.

**Results:**

A total of 33 patients participated in the study. The BDI-II results indicated that 91% (n=30) of the patients presented a minimal level. In BAI, 94% (n=31) of the patients demonstrated minimal level of anxiety symptoms. WHOQOL-BREF showed a perception of quality of life considered good in all domains. The EMEP data have registered a problem-focused coping strategy. According to MINI, a single case of major depressive episode, current and recurrent was recorded.

**Conclusion:**

Although most participants in the sample had symptoms of depression and anxiety, only one patient was identified with moderate symptoms in both domains. The most used strategy was coping focused on the problem. Patients have classified the perceptions of quality of life as 'good', pointing out satisfaction with their health.

**Table:** 

Abbreviations, acronyms & symbols	
BAI	= Beck Anxiety Inventory
BDI-II	= Beck Depression Inventory
EMEP	= Escala Modos de Enfrentamento de Problemas (Ways of Coping Scale)
HDL	= High-density lipoprotein cholesterol
HF	= Heart failure
SatePsi	= Sistema de Avaliação de Testes Psicológicos (Psychological Testing System)
WHOQOL-BREF	= World Health Organization Quality of Life-BREF

## INTRODUCTION

Data from World Health Organization^[1]^ indicate that in 2012 alone, 17.5 million people died due to cardiovascular diseases, with 80% of deaths occurring in middle- and low-income countries.

As the heart is essential for maintenance of life, issues related to this organ are closely associated with behavior and emotions. When a cardiopathy occurs, a complex interaction of variables interferes in the progression and patient's recovery^[2]^.

The chronic character of heart diseases is associated with threats and uncertainties "which relate to psychic suffering, psychosocial integrity, social disadvantage, physiological incapacities, uncertainty of treatment success, possibility of rejection leading to death"^[3]^.

A significant number of studies have found a relation between mood disorders, anxiety and heart disease. The presence of depressive symptoms in patients with heart disease doubles the risk of death associated with cardiovascular diseases. Thus, rehabilitation of a patient with heart disease should not only address physical and physiological functions, but also cognitive, adaptive and psychological functioning, especially if, in the post-operative period, the patient receives a new heart, such as cardiac transplantation^[4]^.

In more severe heart diseases, such as refractory heart failure (HF), cardiac transplantation is recognized as the best therapeutic strategy. It is therefore indicated for those patients with worse prognosis and without alternatives of clinical treatments^[5,6]^.

Evaluation and follow-up of interdisciplinary teams are fundamental for candidates and patients who underwent heart transplant. From a psychosocial point of view, stress, negative emotions, coping resources, lifestyle and other factors can have a significant impact on the recovery and rehabilitation of these patients. The presence of mental disorders in these patients "have a negative and direct repercussion on adherence, necessary lifestyle modifications and the discipline imposed by the treatment protocol"^[5]^.

After cardiac transplantation, concerns regarding the condition of being a post-transplant patient require adaptations in lifestyle, including, for example, infection care, properly balanced diet, weight maintenance, strict and continued administration of immunosuppressive drugs, adherence to the team's advices for maintenance of well-being, and reduction of complications, especially in the early months.

Approaching the psychological aspects of these patients becomes necessary throughout the entire process, mainly in patient discharge and longitudinal follow-up^[3]^. A successful cardiac transplantation not only prolongs life, but also optimizes the patient's psychosocial functioning^[2,7]^.

Considering the importance of psychosocial factors in heart disease and the maintenance of adaptive resources after cardiac surgeries, this study aims to identify, in patients who underwent cardiac transplantation, the presence of mental disorders and symptoms of depression and anxiety; to evaluate the perception of quality of life and coping strategies after heart transplantation; to correlate variables and compare them in relation to gender, age group and length of time after transplantation.

## METHODS

**Fig. 1:**
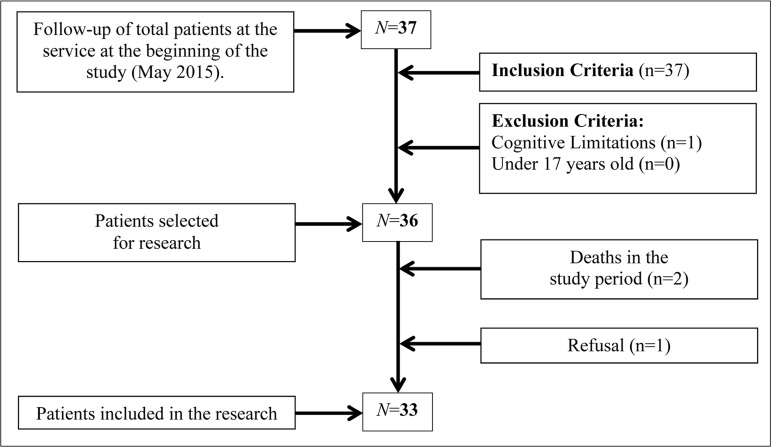
Systematic representation of the screening method and sample selection.

Cross-sectional, population-based descriptive study, with quantitative analysis. A total of 33 patients under follow-up after heart transplantation were enrolled in the Heart Transplant Service of the Hospital de Base de São José do Rio Preto, aged 17 years and over, regardless of class or gender. All patients attending the service (N=37) were invited at the start date of collection, regardless of the length of time after transplantation, that was between 4 and 14 years (Figure 1).

One of them presented cognitive limitations, one refused to participate due to lack of time to respond to the questionnaires and two died before being evaluated. The small sample size is justified by the relatively low frequency of heart transplants in the unit and the deaths. The study only included patients who underwent heart transplantation at the Hospital de Base de São José do Rio Preto, state of São Paulo.

The instruments used in the research protocol are approved for use by SatePsi (Sistema de Avaliação de Testes Psicológicos, Psychological Testing System) and regulated by the Federal Council of Psychology; structured and validated in the Brazilian version; with quick and simple administration and interpretation; with reduced cost; seen in similar studies; and easy to understand.

Based on literature, its guidelines manuals and practice, the average time of administration of all the instruments was 1 hour and 30 minutes^[8-11]^. A pilot protocol was performed in order to ascertain the administration time of the instruments and to define the order of administration of the questionnaires. Within these criteria, were selected to compose the protocol:

- Sociodemographic questionnaire, created by the researcher to collect sociodemographic data. The instrument has nine questions about sex, age, education, marital status, number of children, household size or number of close family and friends, employment status, length of time after transplant and cardiopathy;
- Beck Depression Inventory (BDI-II), a Brazilian version of an instrument whose objective is to measure the intensity of somatic and cognitive-affective symptoms of depression in adults and adolescents from 13 years old^[10]^;
- Beck Anxiety Inventory (BAI), a self-report scale that measures the presence and intensity of anxiety symptoms^[9]^;
- WHOQOL-BREF (World Health Organization Quality of Life-BREF), instrument of the World Health Organization that assesses quality of life^[12]^;
- Ways of Coping Scale (Escala Modos de Enfrentamento de Problemas, EMEP), an instrument that evaluates four domains: Problem-Focused Coping, Emotion-Focused Coping, Seeking Social Support and Seeking Religious Practices, identifying religious thoughts and behaviors as coping and ways of coping and stressor management^[11]^;
- MINI International Neuropsychiatric Interview, a brief standardized diagnostic interview (15-30 minutes) compatible with the DSM-IV and ICD-10 criteria for mental disorders^[8]^.

### Administration Procedures

Participants were asked to fill out the questionnaires on the days of their follow-up visits, which used to happen every six months for most transplant recipients, or while performing some other procedure at the health facility. The data collection took place in a single session for each patient, individually, in the waiting room, in bed or any other place that maintained the secrecy conditions of the collection.

### Data Analysis

Data were analyzed using descriptive and inferential statistics. The normality tests Lilliefors, Spearman's Rank Correlation Coefficient (r) and sample comparisons of non-parametric data with Mann-Whitney test and parametric data with T-Test were used, assuming a significance level *P*≤0.05.

### Ethical Aspects

Project (CAAE: 43844315.1.0000.5415) approved by the Research Ethics Committee of FAMERP (Opinion No. 1.059.672, May 12, 2015).

## RESULTS

The study sample consisted of 33 cardiac transplant patients with predominant male distribution (67%, n=22), incomplete elementary school (48%, n=16), married (70%, n=23), retired (85%, n=28), living with spouse and/or children (76%, n=25) and living with three or four people (61%, n=20).

**Fig. 2:**
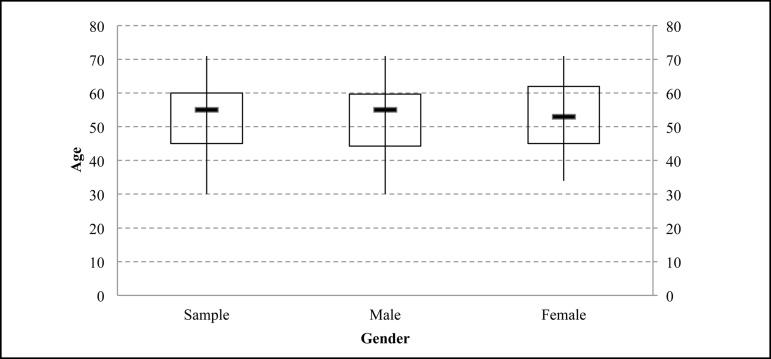
General age and sex ratio of the sample participants.

Although the age of the sample varied between 30 and 71 years, the age range of the majority of study participants was concentrated between 30 and 60 years (MD=55) (Figure 2).

In the comparison by sex, women presented higher ages. Among the male participants, the age range varied between 30 and 71 years, with concentration between 44 and 59 years (MD=55). Among women, the age varied between 34 and 71 years, with concentration in the range of 45 to 62 years (MD=53).

**Fig. 3:**
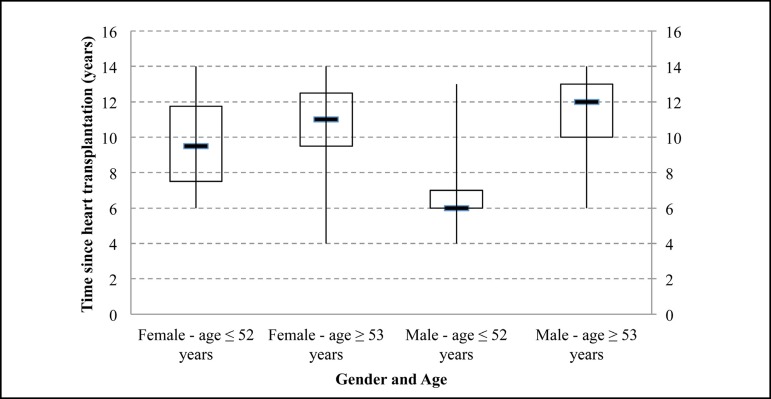
Length of time after heart transplantation by gender and age.

The length of time after transplantation indicates a minimum of four years and a maximum of 14 years for both sexes (Figure 3). Differences could be observed only in concentration by age. The time since heart transplantation in the female sample, aged 52 years or less, was between 7 and 12 (MD=9). In women aged 53 years or over, they had a concentration between 9 and 12 (MD=11). Among men, those up to 52 years old presented a concentration between 6 and 7 (MD=6) and those aged 53 years and over, concentration between 10 and 13 (MD=12).

Among the psychosocial variables, symptoms of depression and anxiety, perceived quality of life and coping strategies were evaluated.

Assessment of depression symptoms indicated that 91% (n=30) of the patients had minimal symptoms, 6% (n=2) mild symptoms and 3% (n=1) moderate symptoms. When compared by gender, 95% (n=21) of the men presented minimal symptoms and 5% (n=1) light symptoms, without occurrence of moderate and severe symptoms; 82% (n=9) of the women presented minimal symptoms and 18% (n=2) had mild or moderate symptoms, without serious events. Data from MINI revealed that 3.03% (n=1) presented diagnostic criteria for depression. The instrument did not identify the presence of other mental disorders.

**Fig. 4:**
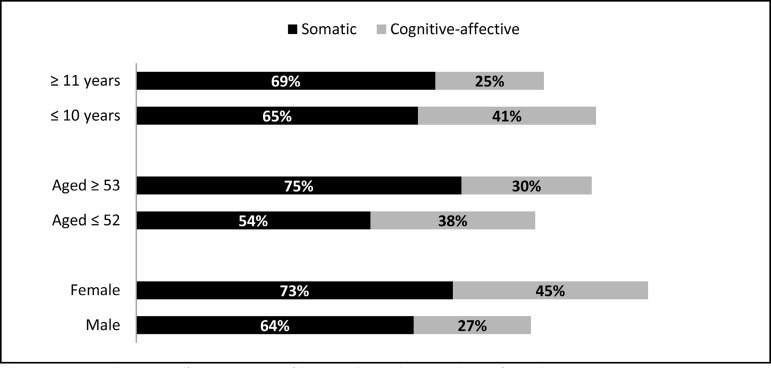
Somatic and cognitive-affective symptoms of depression by gender, age and time of transplantation.

Figure 4 shows the percentage of occurrences of somatic and cognitive-affective symptoms of depression after cardiac transplantation, as well as the comparison by gender, age group and time since transplantation.

The assessment of anxiety symptoms indicated that 94% (n=31) of the symptoms experienced by patients focused on the minimum level. Mild and moderate levels were also identified (6%, n=2). Minor symptoms were presented by 95% (n=21) of males and 91% (n=1) of females; 5% (n=1) of the male sample had mild symptoms and 9% (n=1) of the female sample had moderate anxiety symptoms. No serious events of anxiety symptoms have been identified in patients.

**Fig. 5:**
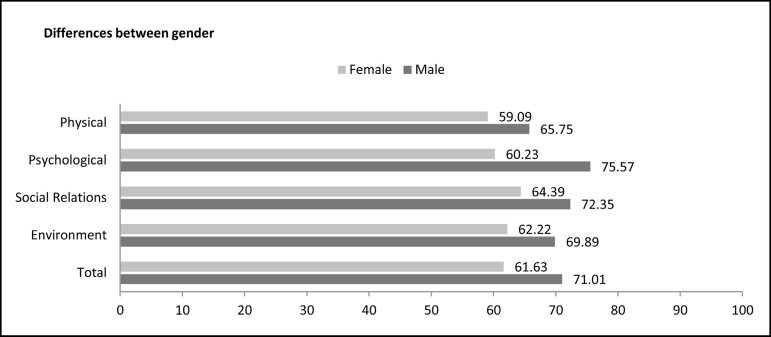
Domains of quality of life after heart transplantation by gender.

The perception of quality of life and satisfaction in physical, psychological, social and environmental domains was assessed (Figure 5). The values are presented on a scale of 0 to 100: 0-20 (very bad), 21-40 (bad), 41-60 (neither bad nor good), 61-80 (good), and 81-100 (very good).

In the overall sample, the data indicated 'good' perceptions in all domains. Women assessed all quality of life domains as 'good', except the physical domain, classified as 'neither bad nor good'. Men, however, have revealed 'good' perceptions in all domains evaluated. No domain was considered 'very good', 'bad' or 'very bad'. Significant differences between the sexes were found in the psychological domains (*P*=0.0071) and in the environmental satisfaction (*P*=0.0387), with higher results for men.

Regarding the coping methods used by patients to deal with stressors, high scores were obtained for the use of problemoriented strategies, followed by a focus on religiosity or fanciful thinking and social support. Less frequent strategies (lower scores) were found for emotion-focused coping.

Significant differences were found in the domain of coping focused on religiosity or fanciful thinking in comparison with age group. Older patients (53 years or older) had higher scores on coping focused on religion or fanciful thinking (*P*=0.039).

Table 1 presents the correlations between ways of coping with problems and symptoms of depression, anxiety and quality of life. Table 2 presents positive (proportional) or negative (inversely proportional) aspects of the significant correlations shown in Table 1.

**Table 1:** Correlation between ways of coping with problems, symptoms of depression, anxiety and quality of life of patients after heart transplantation.

Correlation a*P*-value	Coping	Focus on problem	Focus on emotion	Focus on religiosity	Focus on social support
Anxiety symptoms	0.8879	0.7094	0.1649	0.0592	0.5463
Depression symptoms	0.0069**	0.0021**	0.0571	0.8526	0.0104*
Perception of QOL	0.0026**	≤ 0.0001****	0.0108*	0.2415	0.0092**
Satisfaction with health	0.023*	0.0002***	0.0414*	0.2149	0.1153
Physical domain	0.011*	≤ 0.0001****	0.0027**	0.0773	0.1262
Psychological domain	0.0002***	≤ 0.0001****	0.0073**	0.4103	0.002**
Social relations	0.0002***	≤ 0.0001****	0.002**	0.513	0.0002***
Environment	0.0011**	≤ 0.0001****	0.0132*	0.3212	0.0039**

QOL=Quality of life;

a Spearman Test^®^

* P≤0.05;

** P≤0.01;

*** P≤0.001;

**** P≤0.0001

**Table 2:** Significant positive and negative correlations between ways of coping with problems, symptoms of depression, anxiety and quality of life of patients after heart transplantation.

Correlation *Value *(r)*	Coping	Focus on problem	Focus on emotion	Focus on social support
Depression symptoms	–0.4616	–0.5157	*ns*	–0.4402
Perception of QOL	+ 0.5067	+ 0.6456	–0.4379	+ 0.4463
Satisfaction with health	+ 0.3947	+ 0.6065	–0.3569	*ns*
Physical domain	+ 0.4369	+ 0.7544	–0.5055	*ns*
Psychological domain	+ 0.6006	+ 0.8461	–0.4582	+ 0.5183
Social relations	+ 0.6009	+ 0.7194	–0.5178	+ 0.6011
Environment	+ 0.5433	+ 0.6912	–0.4269	+ 0.4891

QOL= Quality of Life; ns=not significant

* Spearman Test (r); (-) negative significant correlations; (+) positive significant correlations

When coping strategies were correlated with depression symptoms, there was a significant negative correlation between the two variables (r=-0.4616; *P*≤0.01).

Significant positive correlations were found between coping strategies and perceived quality of life (r=0.5067; *P*≤0.01), levels of health satisfaction (r=0.3947; *P*≤0.05), physical domain (r=0.4369; *P*≤0.05), psychological domain (r=0.6006; *P*≤0.001), social relations (r=0.6009; *P*≤0.001) and environment (r=0.5433; *P*≤0.01).

Significant negative correlation (r=-0.5157; *P*≤0.01) was observed between problem-focused coping and symptoms of depression. Significant positive correlations were observed between problem-focused coping and perceived quality of life (r=0.6456; *P*≤0.0001), health satisfaction (r=0.6065; *P*≤0.001), physical domain (r=0.7544; *P*≤0.0001), psychological domain (r=0.8461; *P*≤0.0001), social relations (r=0.7194; *P*≤0.0001) and environment (r=0.6912; *P*≤0.0001).

Significant negative correlations were identified between confrontation with emotion-focused coping and perceived quality of life (r=-0.4379; *P*≤0.05), health satisfaction (r=-0.3569; *P*≤0.05), physical domain (r=-0.5055; *P*≤0.01), psychological domain (r=-0.4582, *P*≤0.01), social relations (r=-0.5178; *P*≤0.01) and environment (*r*=-0.4269; *P*≤0.05).

Significant correlations were also observed for coping with focus on social support. When correlated with symptoms of depression, a negative correlation was observed (r=-0.4402, *P*≤0.05). Significant positive correlations were observed with perceived quality of life (r=0.4463, *P*≤0.01), satisfaction with psychological domain (r=0.5183, *P*≤0.01), social relations (r=.6011, *P*≤0.001) and environment (r=0.4891, *P*≤0.01).

## DISCUSSION

Data of 33 patients' psychological assessment was analyzed. The sample size corresponds to the number of patients evaluated from May 2015 and April 2016 and it is due to the low frequency of heart transplantation at the Institution.

Sociodemographic characteristics similar to the observed in this study were found in the literature. A study carried out in a hospital in Belo Horizonte, which investigated the demographic and epidemiological profile of patients undergoing a heart transplant, observed a predominance of male patients, living in cohabitation, with incomplete primary education and retirees. The average age range of 53 years can also be observed in other studies on the subject^[2,7,13,14]^.

The predominantly male profile of the participants is justified because of the advanced age and because male gender is associated with the number and intensity of risk factors for heart diseases. Studies analyzing the prevalence of risk factors by sex have found that smoking, uncontrolled blood pressure, high blood cholesterol, low levels of high-density lipoprotein cholesterol (HDL), and elevated levels of triglycerides are significantly more prevalent in men than in women^[1]^.

When observing the low levels of symptoms of depression and anxiety, we can draw a parallel with studies that monitored and evaluated these same variables in patients in the waiting list and up to 12 months after transplantation, which also revealed a decrease in these symptoms. During the wait for transplantation, 32.1% of the patients had psychological disorders. After surgery, the indices had a decrease of 21.3%^[15]^. This reduction is attributed to a better perception of quality of life related to physical aspects, return to activities of daily living, to work and, above all, to increased survival. The literature also shows that these patients dedicate a greater focus to future planning, especially regarding help and social support needs, and this focus is the cause for reduced symptoms of anxiety, depression and good levels of quality of life, approaching of mental health levels of the general population^[16]^.

However, it is important to evaluate symptoms of depression in these patients and to discriminate them in terms of their characteristics, *i.e.*, somatic or cognitive. Studies that have reported the occurrence of depression symptoms associated with heart disease have raised questions about the validity of evaluating depression symptoms with self-report questionnaires in the context of acute or chronic medical conditions. Physical symptoms after heart surgeries resemble the somatic symptoms of depression. Thus, an assessment of depression symptoms in these patients should discriminate among somatic symptoms of depression and symptoms of the disease itself^[17,18]^.

According to Almeida et al.^[19]^, typical physiological aspects of post-transplantation influence cardiovascular rehabilitation after transplantation. The authors investigated complications following cardiac transplantation and pointed out some physical symptoms that arise from the clinical presentation: cardiac denervation, lower heart rate response to exercise, increased resting heart rate and sympathetic tone, use of immunosuppressive drugs with effects on muscle, possibility of cardiac cachexia and physical deconditioning that may be of long duration, arterial hypertension, subjective fatigue and others.

Depression is associated with somatic and behavioral changes. Thus, the instrument used to evaluate symptoms of depression, BDI-II, divided these symptoms reported by the patients into Cognitive-affective and Somatic-affective. The second factor, also known as 'somatic', includes: lack of energy, changes in sleeping patterns and appetite, irritability, difficulty concentrating and tiredness or fatigue^[18,20]^.

Common symptoms after cardiac surgery include fatigue or loss of energy, changes in sleep, changes in appetite, and "may be misinterpreted by healthcare providers, researchers or patients as mood-related"^[18]^, precisely because they resemble the somatic symptoms of depression.

The findings of this study resemble those of other studies, as pointed out by Thombs et al.^[18]^. The same instrument used in this study to measure symptoms of depression (BDI-II), when used in patients with heart diseases, revealed that 50% to 75% of the patients report occurrence of somatic symptoms, which may be responsible for up to 10 points in the total score of depression symptoms. This factor should be observed by the professional who will evaluate patients in these situations, since there is considerable practical difficulty in determining if specific somatic symptoms, such as fatigue and change of appetite, are a result of the patient's depression or medical condition.

Regarding the quality of life of the sample, despite the possible adverse outcomes due to the surgical process, such as infections, hospitalization and frequent hospitalizations, lifestyle adjustments and frequent use of medications, the data showed a good perception of general quality of life, with better scores for the psychological domain, followed by social domain, environment and physical domain. Vasconcelos et al.^[6]^ suggest that cardiac transplantation is perceived by patients as a possibility to restore their health conditions, being one of the reasons for good perceptions of quality of life. "Even with all the difficulties, organ receptors were happy, grateful and victorious for surviving after heart transplantation"^[6]^.

Literature shows similar data to those compiled in this study regarding quality of life. Czyzewski et al.^[14]^ point to the increase of good perception of the quality of life in patients after cardiac transplantation. Studies indicate that quality of life before heart transplantation is correlated with subjective perception of health, while in post-transplantation the perception of quality of life is directly related to the level of satisfaction of the patient with his/her health status, which is one of the main factors that allow the increase of indices^[14]^.

In order to cope with the post-transplantation situation, aiming at reducing stress, patients in this study used more problem-focused coping than emotion-focused coping, the latter less strategy used.

Collected data resemble the Seidl et al.^[11]^ observations, when they affirm that the results regarding the use of emotion-oriented strategies are smaller in people whose stressor agent is a health problem. Authors discussing emotion-focused coping postulate that the use of this strategy expresses feelings of guilt about oneself and the other, as well as avoidant behaviors concomitant with negative emotions, leading one to suppose that, in the case of higher scores, it would also be possible to find relevant psychological difficulties.

Researches have found similar results to those obtained in this study regarding the strategies used by each of the sexes. Men use problem-focused strategies and women use religious/fantasy practices^[21]^.

Findings on coping in women showed higher averages oriented to religious practices, followed by focus on the problem, seeking social support and, finally, focus on emotion. For some women, the disease presents itself as a "bargaining process with God, of life and health, for others, an opportunity to acquire new values, rethink life and value the spiritual side, which is put into practice after the disease"^[21]^. This information influences the results obtained in the studies.

When correlating coping strategies with depression, levels were inversely proportional. The more the patient used coping strategies, the smaller the symptoms of depression. Kroemeke^[17]^ states that this correlation is expected, since patients experience greater or lesser symptoms of depression according to their ability to evaluate events, their coping strategies and their efforts made available to act. The author also emphasizes that patients with low depression symptoms have a more positive perception of the disease and show greater adaptive resources facing the situation.

Patients with good indexes of coping strategies had better perceptions of quality of life, greater satisfaction with health and with physical, psychological, social and environmental aspects. Studies have shown that patients achieve significant improvements in their quality of life when they have greater social, family and spiritual support^[6]^.

The more patients use problem-oriented coping strategies, the lower the symptoms of depression. Bonanno et al.^[22]^ concluded that patients with low rates of depressive symptoms are more likely to deal with problem-focused strategies and less likely to use emotion-focused strategies, which resembles the findings of this study. Nunes et al.^[23]^ found a significant association between focus on the problem and symptoms of depression, pointing out that this relationship indicates that the greater the focus on the problem, the less likely the presence of depressive symptoms or maybe "the more depressive symptoms, the less likely the adoption of strategies focused on the problem".

Data indicate that the less patients use emotion-oriented coping strategies, the better the perceptions of quality of life and satisfaction with health, physical domain, psychological domain, social relations and environment. Nunes et al.^[23]^, who investigated the coping methods most used in the process of illness, revealed similar data. The authors found that the emotionbased coping method is positively correlated with the presence of anxious and depressive symptoms, *i.e*., the more patients focused on emotion, the greater the symptoms of anxiety and depression, leading to impairments in quality of life.

The numbers of this study indicate that the more focus on social support the patient presented, the better his/her perception of quality of life and his/her satisfaction with the psychological domain, with social relations and with the environment.

Data found in literature revealed that, besides social support being considered a factor of protection and health promotion, its positive effects are associated with different types of support offered by the family, being able to be both emotional and functional, *i.e*., support related to activities of daily living. Thus, social support allows a more adequate management of emotions, affective, cognitive and feedback orientations, helping to increase the patient's adaptive skills^[24]^.

In face of the high prevalence of risk factors and cardiovascular diseases in men and post-transplanted male predominance in this sample, all the results were more significant in men. This feature has a straight link with the results of this study. At the same time that we observed that the majority was male patients, we also observed lower levels of depression, anxiety (and other psychological disorders), as well as better perceptions of quality of life, satisfaction with health and problem-focused coping, which means more adequate ways of facing the post-surgical and follow-up conditions. Although more vulnerable to risk factors and cardiovascular diseases, men are likely to face more objectively a situation than women. Women used more coping focused on religious thoughts, which is more subjective; thus, obtaining inferior results.

Thus, pre-transplant psychological preparation, as well as follow-up after transplantation, are required. The type of post-transplant follow-up and its impact still need to be better assessed. Pfeifer & Ruschel^[25]^ concluded that heart transplant preparation was associated with better coping indexes when compared to post-transplant follow-up.

Cardiac transplantation, besides being the most effective therapy for patients with refractory heart disease, also represents the possibility of improvements in quality of life. The surgical procedure, concomitant with a psychological evaluation, pretransplant psychological preparation and post-transplantation follow-up, allow the development of coping strategies, improving symptoms of depression and anxiety, and directly influencing the perception of quality of life and levels of satisfaction with health. Thus, interdisciplinary teams are needed to ensure favorable conditions for recovery, adherence to treatment and solving problems that arise in the course of procedures.

Regarding the psychologist's performance in health teams, individual and group visits are suggested. While the individual visit aims psychic reorganization, readaptation and resignification in face of the new perspective of health, group care creates the possibility of contact with other transplant patients. This contact, often performed in waiting room, places patients and family members with information and appropriate models of coping.

Although many studies have evaluated these variables separately, we did not find any showing correlation or association between these variables in post-transplanted patients. Therefore, this study provides contributions to clinical practice and psychological interventions with these patients. According to the results, psychological interventions should focus on problem-focused coping, enabling patients to have lower rates of anxiety, depression and better perceptions of quality of life and satisfaction with their health.

This study also alerts clinicians and researchers that depression assessment through somatic symptoms should be performed with caution, since physical symptoms resulting from heart transplant may resemble depression somatic symptoms.

Some limitations of this study can be pointed out. Even if the design has allowed obtaining information and evaluations of the variables of interest, the cross-sectional method does not allow the definition of causal associations between domains, since, in the case of an interruption in time, the possible determinants and outcomes are seen at the same time, preventing the use of criteria of causes.

This study design also does not assure inferences about the longitudinal impact of the studied variables. There is a need for a control group and broadening of knowledge about the development of aspects related to symptoms, perceptions and coping, as well as the emotional responses of patients who underwent transplantation in the short, medium and long-term.

The daily life of a patient undergoing heart transplantation is characterized by new situations that require adaptations of the family and social network. Including family and the social group in future research is relevant to a more comprehensive understanding of the phenomenon in question, since this study was limited to the patient's perceptions.

## CONCLUSION

Based on the outcomes of this study, we can conclude that patients who underwent cardiac transplantation showed good perceptions of quality of life in all domains, even with minimal, mild and moderate levels of symptoms of depression and anxiety. Patients who underwent transplantation had more somatic symptoms of depression. Thus, it is important to distinguish between cognitive and somatic symptoms when evaluating depression in patients with chronic diseases, since somatic symptoms may be due to illness rather than a depressive process itself.

Despite the use of all coping styles, there was predominance of problem-focused coping and less use of emotion-oriented strategies that are probably associated with the positive indices of perception of quality of life and the low occurrences of depression and anxiety in the sample.

**Table:** 

Authors' roles & responsibilities	
FBT	Drafting the work or revising it critically for important intellectual content; acquisition, analysis, or interpretation of data for the work; final approval of the version to be published
MCOSM	Drafting the work or revising it critically for important intellectual content; acquisition, analysis, or interpretation of data for the work; final approval of the version to be published
YLWS	Drafting the work or revising it critically for important intellectual content; acquisition, analysis, or interpretation of data for the work; final approval of the version to be published
CMWR	Drafting the work or revising it critically for important intellectual content; acquisition, analysis, or interpretation of data for the work; final approval of the version to be published
